# Long-term exposure to ambient PM_2.5_ and its components on menarche timing among Chinese adolescents: evidence from a representative nationwide cohort

**DOI:** 10.1186/s12889-024-18209-2

**Published:** 2024-03-05

**Authors:** Danting Li, Jingyuan Xiong, Guo Cheng

**Affiliations:** 1 Department of Nutrition and Food Safety, West China School of Public Health and West China Fourth Hospital, Sichuan University, Chengdu, Sichuan China; 2 Healthy Food Evaluation Research Center, West China School of Public Health and West China Fourth Hospital, Sichuan University, Chengdu, Sichuan China; 3 Laboratory of Molecular Translational Medicine, Center for Translational Medicine, Key Laboratory of Birth Defects and Related Diseases of Women and Children, Ministry of Education, Maternal & Child Nutrition Center, West China Second University Hospital, Sichuan University, 610041 Chengdu, Sichuan China

**Keywords:** Menarche, Particulate matter, PM_2.5_ components, Female adolescents, Pubertal development, Weighted quantile sum model

## Abstract

**Background:**

Ambient air pollutants have been suggested to affect pubertal development. Nevertheless, current studies indicate inconsistent effects of these pollutants, causing precocious or delayed puberty onset. This study aimed to explore the associations between long-term exposure to particulate matter with aerodynamic diameters ≤ 2.5 μm (PM_2.5_) along with its components and menarche timing among Chinese girls.

**Method:**

Self-reported age at menarche was collected among 855 girls from China Health and Nutrition Survey 2004 to 2015. The pre-menarche annual average concentrations of PM_2.5_ and its components were calculated on the basis of a long-term (2000–2014) high-resolution PM_2.5_ components dataset. Generalized linear models (GLM) and logistic regression models were used to analyze the associations of exposure to a single pollutant (PM_2.5_, sulfate, nitrate, ammonium, black carbon and organic matter) with age at menarche and early menarche (< 12 years), respectively. Weighted quantile sum methods were applied to examine the impacts of joint exposure on menarche timing.

**Results:**

In the adjusted GLM, per 1 µg/m^3^ increase of annual average concentrations of nitrate and ammonium decreased age at menarche by 0.098 years and 0.127 years, respectively (all *P* < 0.05). Every 1 µg/m^3^ increase of annual average concentrations of PM_2.5_ (OR: 1.04, 95% CI: 1.00-1.08), sulfate (OR: 1.23, 95% CI: 1.01–1.50), nitrate (OR: 1.23, 95% CI: 1.06–1.43) and ammonium (OR: 1.32, 95% CI: 1.06–1.66) were significantly positively associated with early menarche. Higher level of joint exposure to PM_2.5_ and its components was associated with 11% higher odds of early menarche (*P* = 0.04). Additionally, the estimated weight of sulfate was the largest among the mixed pollutants.

**Conclusions:**

Long-term exposure to PM_2.5_ and its components could increase the risk of early menarche among Chinese girls. Moreover, sulfate might be the most critical components responsible for this relationship. Our study provides foundation for targeted prevention of PM_2.5_ components.

**Supplementary Information:**

The online version contains supplementary material available at 10.1186/s12889-024-18209-2.

## Introduction

Age at menarche earlier than 12 years is normally classified as early menarche [1], and it has been linked with later adverse health outcomes in life, including breast cancer [2], type 2 diabetes [3], cardiovascular diseases [4] as well as psychological disorders [5, 6]. Besides, girls who experienced early menarche are prone to have risky sexual behaviors such as early sexual initiation and sexual transmission infections [7]. The median age at menarche had consistently declined from 13.37 years in 1985 to 12.00 years in 2019 among Chinses girls, which indicated a conspicuous trend of early puberty onset [8].

Evidence has shown that age at menarche can be determined by genetic and non-genetic factors [9]. In spite of the greater role of genetic factors, a variety of studies concentrate on non-genetic factors that are modifiable like socioeconomic status, nutrition status and environmental conditions [10–13]. It is of major public health relevance if these factors could be controlled and hence improving health and life quality in adulthood.

Ambient air pollution, among many environmental factors, is postulated to be a risk factor for abnormal pubertal development. Accumulating studies have suggested that endocrine-disrupting chemicals (EDCs) in the atmosphere can disturb biosynthesis of hormones and regulate hormone levels, resulting in alteration of puberty timing [14, 15]. Although the potential mechanisms are still not pinpointed, recently, a few studies have unveiled that air pollutants such as particulate matter (PM), nitrogen dioxide (NO_2_), sulfur dioxide (SO_2_) or ozone (O_3_) might be associated with puberty timing [12, 16–19]. However, these studies were grounded on various assessment of pubertal development (e.g., Tanner stage, concentration of sex hormone, self-reported age at menarche) and yielded heterogeneous results in general. It is notable that none of the existing studies have explored the associations of PM with aerodynamic diameters ≤ 2.5 μm (PM_2.5_) and its components with menarche timing to date.

Therefore, using data from a national representative cohort, this study aimed to examine the associations of single and joint exposure to PM_2.5_ and its major components (sulfate, nitrate, ammonium, black carbon [BC] and organic matter [OM]) with menarche timing among female adolescents in China.

## Materials and methods

### Study population

The present study used data from the recent five waves (2004, 2006, 2009, 2011 and 2015) of China Health and Nutrition Survey (CHNS), an open longitudinal cohort study initiated in 1989. Details on the study protocol have been described elsewhere [20]. Briefly, by using a multistage, random cluster sampling method, nationally representative information on economy, socio-demography, public resources, nutrition, and health indicators was collected in 15 provinces and municipalities from China. CHNS is a community, household based study and 7,200 households were involved in overall surveys. All household members provided written informed consent in the survey and could join or withdraw from the study at any survey wave. This study was approved by the Institutional Review Board of the University of North Carolina at Chapel Hill and the National Institute of Nutrition and Health, Chinese Center for Disease Control and Prevention.

**Fig. 1:**
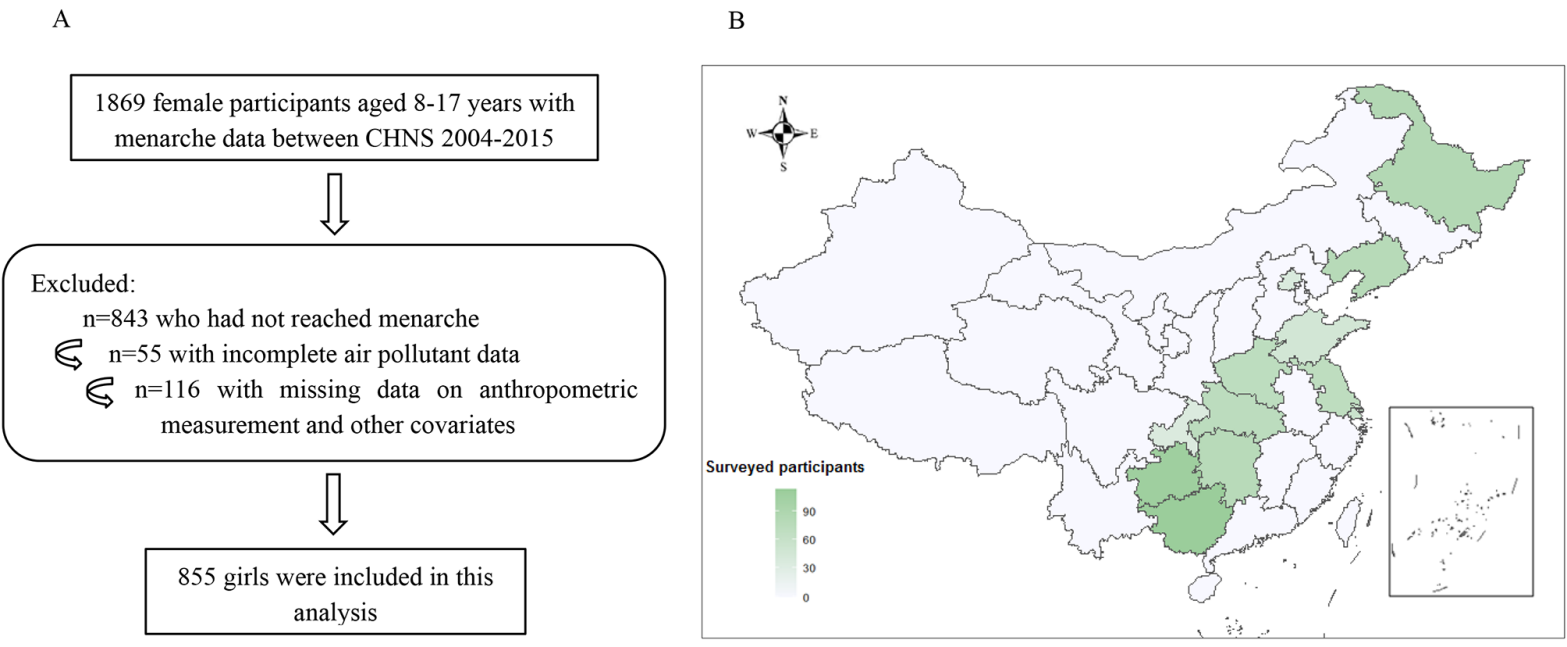
Flow chart and spatial distributions of population density for the study sample. **A**: Flow chart for the study sample; **B**: The spatial distributions of population density and surveyed participants.

In total, 1869 female participants aged 8–17 years completed the questionnaire on menarche and they were interviewed face-to-face by trained investigators at their home. We excluded participants who had not reached menarche (*n* = 843) and those who had missing data on air pollution (*n* = 55). 116 participants with incomplete data on anthropometric measurement and other covariates were further precluded. Finally, the current analysis was based on a sample of 855 (Fig. 1A). The spatial distributions of population density and surveyed district/county were shown in Fig. 1B.

### Menarche timing

Girls aged 8 years or older and/or their guardians were required to complete the questionnaire about menstrual status by answering the questions “Have you already experienced menstruation?” and “How old were you when you had your first menstrual period?” If the girls had not reached menarche during the survey time, the second question was neglected. For those who reported discordant menarcheal ages in different survey waves, only the first provided menarcheal age in the panel data were adopted for analysis to reduce potential recall bias. Prior literature generally defined age at menarche below 12 years as early menarche, which was also applicable for Chinses girls [1, 8], so the cut-off age for early menarche timing was set to 12 years in this study. Participants who experienced menarche before 12 years were categorized into an early menarche group and the rest was assigned into a normal menarche group (≥ 12 years).

### Exposure assessment

The annual average concentration data of PM_2.5_ and its components (sulfate, nitrate, ammonium, BC and OM) from 2000 to 2014 were derived from a well-validated PM_2.5_ component prediction model at 10-km resolution over China from Tracking Air Pollution in China (TAP, http://tapdata.org.cn/). The TAP PM_2.5_ is estimated based on a two-stage machine learning model coupled with the synthetic minority oversampling technique and a tree-based gap-filling method, and the accuracy of the data was verified on a long-time scale [21, 22]. We retrospectively calculated annual mean concentrations of PM_2.5_ and its components according to their registered address for each participant 1-year prior to the year when they experienced menarche (2-year and 3-year average concentration were utilized in sensitivity analysis). As the data were from CHNS, an open longitudinal survey, only long-term dwellers in regular communities were eligible for the survey. Therefore, we considered the participant included in the current analysis had resided at the registered address over the exposure period. The schematic representation of exposure periods could be seen in Additional file 1: Figure S1.

### Covariates

The covariate information was obtained by structured questionnaire, including demographic information such as age at survey time (years), wave (2004, 2006, 2009, 2011 or 2015), ethnicity (Han or minority), surveyed district (Beijing, Liaoning, Heilongjiang, Shanghai, Jiangsu, Shandong, Henan, Hunan, Hubei, Guangxi, Guizhou or Chongqing), residency (urban or rural), household income (yuan), parental education level (≤ 6, 6∼12, or > 12 years of schooling) and lifestyle information on physical activity (no or regular) and second-hand smoke exposure at home (yes or no). Data on temperature were obtained from the BERKELEY EARTH website (https://berkeleyearth.org/) and annual average temperature (°C) one year before the menarche onset was calculated according to the residence of the participants.

Anthropometric measurements were conducted by well-trained health workers according to the standard procedures [23]. Height was measured by a portable wall-mounted metal tape to the nearest 0.1 cm without shoes and weight was measured to the nearest 0.1 kg in light, indoor clothing using a precise digital scale [24]. All the measuring instruments were calibrated before use. Body mass index (BMI) was calculated as weight (kg) divided by height squared (m^2^). Age- and sex-specific BMI z-scores were calculated for each participant based on the growth curves for Chinese children and adolescents aged 0∼18 years [25].

### Statistical analysis

Statistical analyses were performed with SAS procedure (version 9.3, 2011, SAS Institute Inc., Cary, NC, USA) and R software (version 4.3.1, R Development Core Team). To compute the group differences of continuous variables, the t-test was used, and for categorical variables, the chi-square test (χ^2^) was applied. Spearman correlation analysis was performed to test the correlation between PM_2.5_ and its components. A *P*-value < 0.05 was considered statistically significant.

#### Single pollutant exposure analysis

Generalized linear regression was used to examine the associations of 1-year average exposure to PM_2.5_ and its components (µg/m^3^) with age at menarche (years), while multiple logistic regression was conducted to assess the associations between these air pollutants and early menarche onset (early menarche = 1, normal menarche = 0). Three models were performed in this study. In model 1 (crude model), no covariates were included. In model 2, wave (survey year), surveyed district, residency, ethnicity, parental highest education level, physical activity and second-hand smoke exposure at home were adjusted. As body size might have potential mediating effect on pollutant-menarche relations, BMI z-score at survey time (continuous variable) was further adjusted in model 3.

#### Multi-pollutant exposure analysis

The potential effects of joint exposure to the mixture (PM_2.5_ and its components) on age at menarche (years) and early menarche (< 12 years) were estimated using weighted quantile sum (WQS) regression models. Of note, WQS regression can evaluate an individual’s overall exposure burden values [26]. The WQS obtains an index (weighted quantile sum for PM_2.5_ and its components) by determining the weights to all exposures categorized into quartiles or more groups and then incorporates that index into the regression model, which in turn yields effect estimation for mixture exposure [27]. The results of WQS regression model included estimated weight (importance) for every single exposure and effect estimation for the weighted linear index, namely, effects of the mixtures. The component in the mixture with higher weight indicated greater impact on outcomes. R package (gWQS) was used to conduct the WQS analysis. In this study, WQS regression models were based on quartiles of PM_2.5_ and its components (sulfate, nitrate, ammonium, BC and OM), classifying 40% of the data as the training set and 60% as the validation set, and performed bootstrap for 100 times. To enhance comparability, the WQS models adjusted for the identical covariates as in the single pollutant analysis.

#### Sensitivity analysis

To evaluate the robustness of the results, we performed sensitivity analysis by using the 2-year and 3-year average exposure to PM_2.5_ and its components prior to the menarche onset as independent variables. The statistical methods and model adjusting strategies in sensitivity analyses were in accordance with that mentioned above. Furthermore, considering that meteorological factors might be potential confounders of the association between PM_2.5_ (components) and menarche timing [28], we additionally included annual average temperature (°C) as a covariate in the statistic models. The other controlling covariates remained the same.

## Results

### Characteristics of participants

**Fig. 2:**
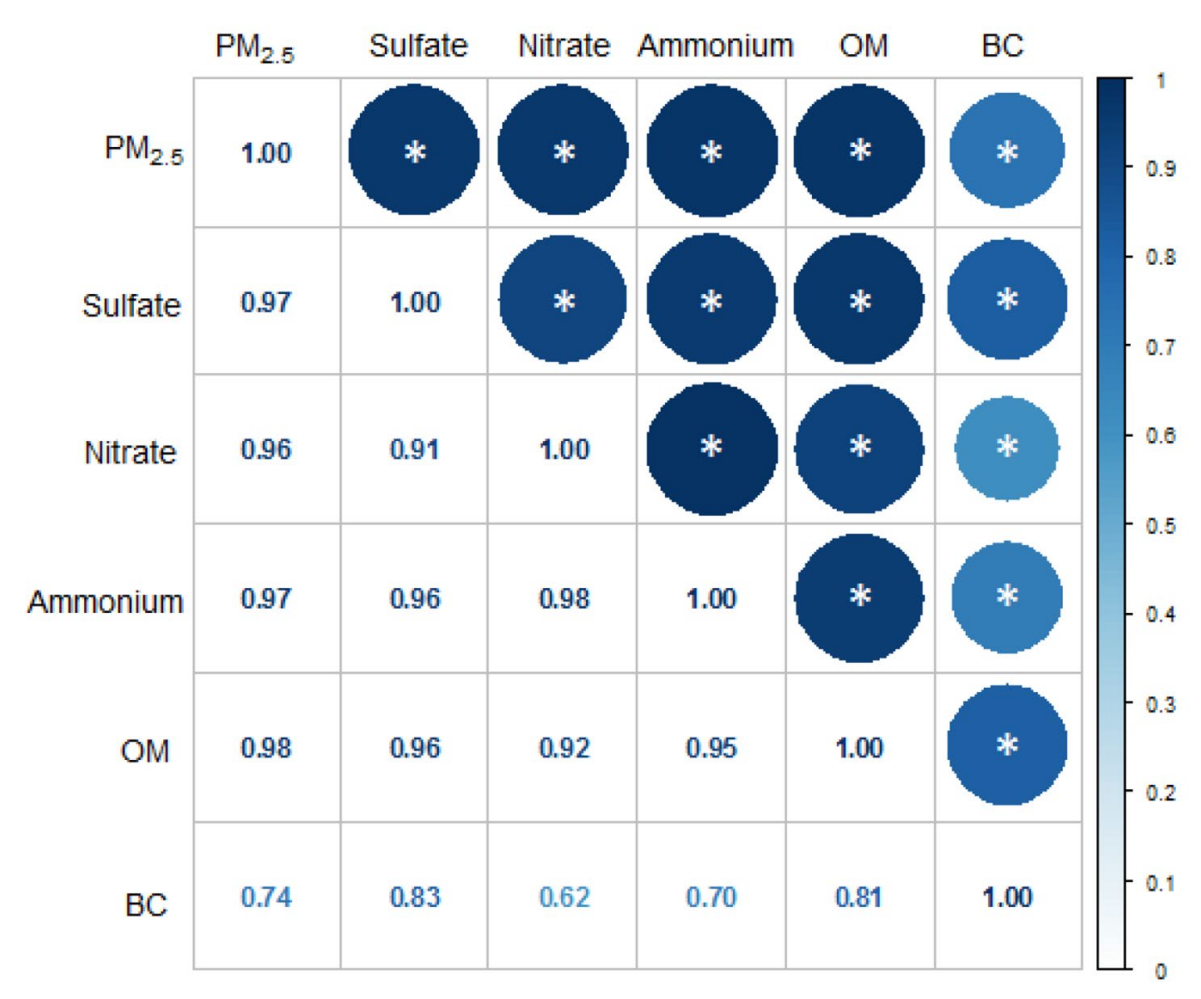
Spearman correlations between PM2.5 and its components. PM_2.5_: particulate matter with aerodynamic diameters ≤ 2.5 μm; OM: organic matter; BC: black carbon. *: *P* < 0.001

Our study sample included 855 girls aged 10–17 years at survey time, 146 (17.1%) of whom were categorized into early menarche (< 12 years) groups (Table 1). Girls who experienced early menarche were more likely to be Han nationality and appeared to engage in physical activity more regularly than their counterparts. The mean BMI z-score of participants in early menarche groups was 0.53 ± 1.38, which was significantly (*P* < 0.001) higher than that of the normal menarche group (-0.02 ± 0.08). Furthermore, the average concentration of PM_2.5_ and its components were higher in early menarche groups (all *P <* 0.05) except for BC. Figure 2 showed high positive correlation between PM_2.5_ and its components, with the Spearman correlation coefficients ranging from 0.62 to 0.98.

**Table 1:** The general characteristics of the study population ^a^

	Total (*n* = 855)	Early menarche group (*n* = 146)	Normal menarche group (*n* = 709)	*P* value
Age at survey time (years)	13.96 ± 1.66	12.14 ± 1.49	14.33 ± 1.43	< 0.001
Wave (survey year)				< 0.001
2004	260 (30.4)	23 (15.8)	237 (33.4)	
2006	131 (15.3)	21 (14.4)	110 (15.5)	
2009	137 (16.0)	33 (22.6)	104 (14.7)	
2011	203 (23.8)	46 (31.5)	157 (22.1)	
2015	124 (14.5)	23 (15.7)	101 (14.3)	
Ethnicity				0.01
Han	742 (86.8)	136 (93.1)	606 (85.5)	
Minority	113 (13.2)	10 (6.9)	103 (14.5)	
Residency				0.9
Urban	317 (37.1)	54 (37.0)	263 (37.1)	
Rural	538 (62.9)	92 (63.0)	446 (62.9)	
Parental highest education level				0.7
≤ 6 years of schooling	186 (21.8)	29 (19.7)	158 (22.3)	
6∼12 years of schooling	582 (68.1)	101 (69.0)	481 (67.8)	
> 12 years of schooling	87 (10.1)	16 (11.3)	70 (9.9)	
Physical activity				0.005
No	583 (68.2)	85 (58.2)	498 (70.2)	
Regularly	272 (31.8)	61 (41.8)	211 (29.8)	
Second-hand smoke				0.5
Yes	596 (69.7)	98 (67.1)	498 (70.3)	
No	259 (30.3)	48 (32.9)	211 (29.7)	
BMI z-score at survey time ^b^	0.07 ± 2.48	0.53 ± 1.38	-0.02 ± 0.08	< 0.001
Age at menarche (years)	12.63 ± 1.28	10.68 ± 0.61	13.03 ± 0.97	< 0.001
Annual average temperature (°C) ^c^	14.50 ± 5.15	15.12 ± 4.54	14.38 ± 5.26	0.1
Air pollutant (µg/m^3^) ^d^				
PM_2.5_	53.08 ± 19.35	56.36 ± 18.55	52.40 ± 19.45	0.01
Sulfate	10.53 ± 3.54	11.19 ± 3.32	10.39 ± 3.57	0.009
Nitrate	9.83 ± 4.51	10.61 ± 4.44	9.67 ± 4.51	0.01
Ammonium	7.42 ± 2.89	7.98 ± 2.82	7.30 ± 2.89	0.006
BC	3.19 ± 0.87	3.27 ± 0.79	3.17 ± 0.89	0.3
OM	13.60 ± 4.12	14.25 ± 3.94	13.47 ± 4.15	0.02

BMI: body mass index; PM_2.5_: particulate matter with aerodynamic diameters ≤ 2.5 μm; BC: black carbon; OM: organic matter

^a^ Data are presented as mean ± standard deviation (SD) for continuous variables and n(%) for categorical variables

^b^ BMI z-scores were calculated according to growth curves for Chinese children and adolescents aged 0∼18 years [25]

^c^ Annual average temperature one year before menarche onset

^d^ Annual average concentration of air pollutant one year before menarche onset

### Single pollutant exposure

After adjusting for wave, surveyed district, residency, ethnicity, parental highest education level, physical activity, second-hand smoke, annual average concentration of nitrate (β = -0.107) and ammonium (β = -0.137) were negatively associated with age at menarche (all *P* < 0.01, Table 2). These negative associations were attenuated, but still remained significant after further adjustment for BMI z-score at survey time in model 3 (Table 2).

**Table 2:** Associations of 1-year average exposure to PM_2.5_ and its components (per 1 µg/m^3^ increase) with age at menarche (year) by generalized linear regression models (*n* = 855) ^a^

Pollutant	Model 1^b^				Model 2^c^				Model 3^d^		
	β	SE	*P* value		β	SE	*P* value		β	SE	*P* value
PM_2.5_	-0.004	0.023	0.1		-0.014	0.008	0.09		-0.013	0.008	0.1
Sulfate	-0.028	0.012	0.02		-0.062	0.042	0.1		-0.062	0.041	0.1
Nitrate	-0.015	0.010	0.1		-0.107	0.032	< 0.001		-0.098	0.031	0.002
Ammonium	-0.034	0.015	0.02		-0.137	0.047	0.004		-0.127	0.046	0.005
BC	-0.095	0.050	0.06		0.113	0.143	0.4		0.063	0.140	0.6
OM	-0.021	0.011	0.05		-0.008	0.034	0.8		-0.012	0.033	0.7

SE: standard error; PM_2.5_: particulate matter with aerodynamic diameters ≤ 2.5 μm; BC: black carbon; OM: organic matter

^a^ Single exposure analysis

^b^ Model 1: crude model, adjusted for no covariates

^c^ Model 2: adjusted for wave, surveyed district, residency, ethnicity, parental highest education level, physical activity, second-hand smoke

^d^ Model 3: adjusted for wave, surveyed district, residency, ethnicity, parental highest education level, physical activity, second-hand smoke, BMI z-score at survey time

For menarche onset, higher exposure to PM_2.5_, sulfate, nitrate, ammonium and OM was significantly related to higher odds of early menarche in model 1 (Table 3). Besides, after controlling for potential covariates, only the first four pollutants presented consistent results. In model 3, for every 1 µg/m^3^ increase of annual average exposure (1-year before menarche onset) to PM_2.5_, sulfate, nitrate and ammonium, the risk of early menarche increased by 4%, 23%, 23%, and 32%, respectively (all *P* < 0.05).

**Table 3:** Associations of 1-year average exposure to PM2.5 and its components (per 1 µg/m^3^ increase) with early menarche (< 12 years) by logistic regression models (*n* = 855) ^a^

Pollutant	Model 1^b^			Model 2^c^			Model 3^d^	
	OR (95% CI)	*P* value		OR (95% CI)	*P* value		OR (95% CI)	*P* value
PM_2.5_	1.01 (1.00, 1.02)	0.02		1.05 (1.01, 1.09)	0.02		1.04 (1.00,1.08)	0.04
Sulfate	1.07 (1.01, 1.12)	0.01		1.25 (1.03, 1.52)	0.03		1.23 (1.01, 1.50)	0.04
Nitrate	1.05 (1.01, 1.09)	0.02		1.27 (1.09, 1.48)	0.002		1.23 (1.06, 1.43)	0.007
Ammonium	1.08 (1.02, 1.15)	0.01		1.37 (1.10, 1.73)	0.006		1.32 (1.06, 1.66)	0.01
BC	1.15 (0.94, 1.42)	0.2		1.19 (0.64, 2.22)	0.6		1.29 (0.68, 2.43)	0.4
OM	1.05 (1.00, 1.09)	0.04		1.11 (0.96,1.29)	0.2		1.11 (0.96, 1.30)	0.2

OR: odds ratio; CI: confidence interval; PM_2.5_: particulate matter with aerodynamic diameters ≤ 2.5 μm; BC: black carbon; OM: organic matter

^a^ Single exposure analysis

^b^ Model 1: crude model, adjusted for no covariates

^c^ Model 2: adjusted for wave, surveyed district, residency, ethnicity, parental highest education level, physical activity, second-hand smoke

^d^ Model 3: adjusted for wave, surveyed district, residency, ethnicity, parental highest education level, physical activity, second-hand smoke, BMI z-score at survey time

### Multi-pollutant exposure

**Fig. 3:**
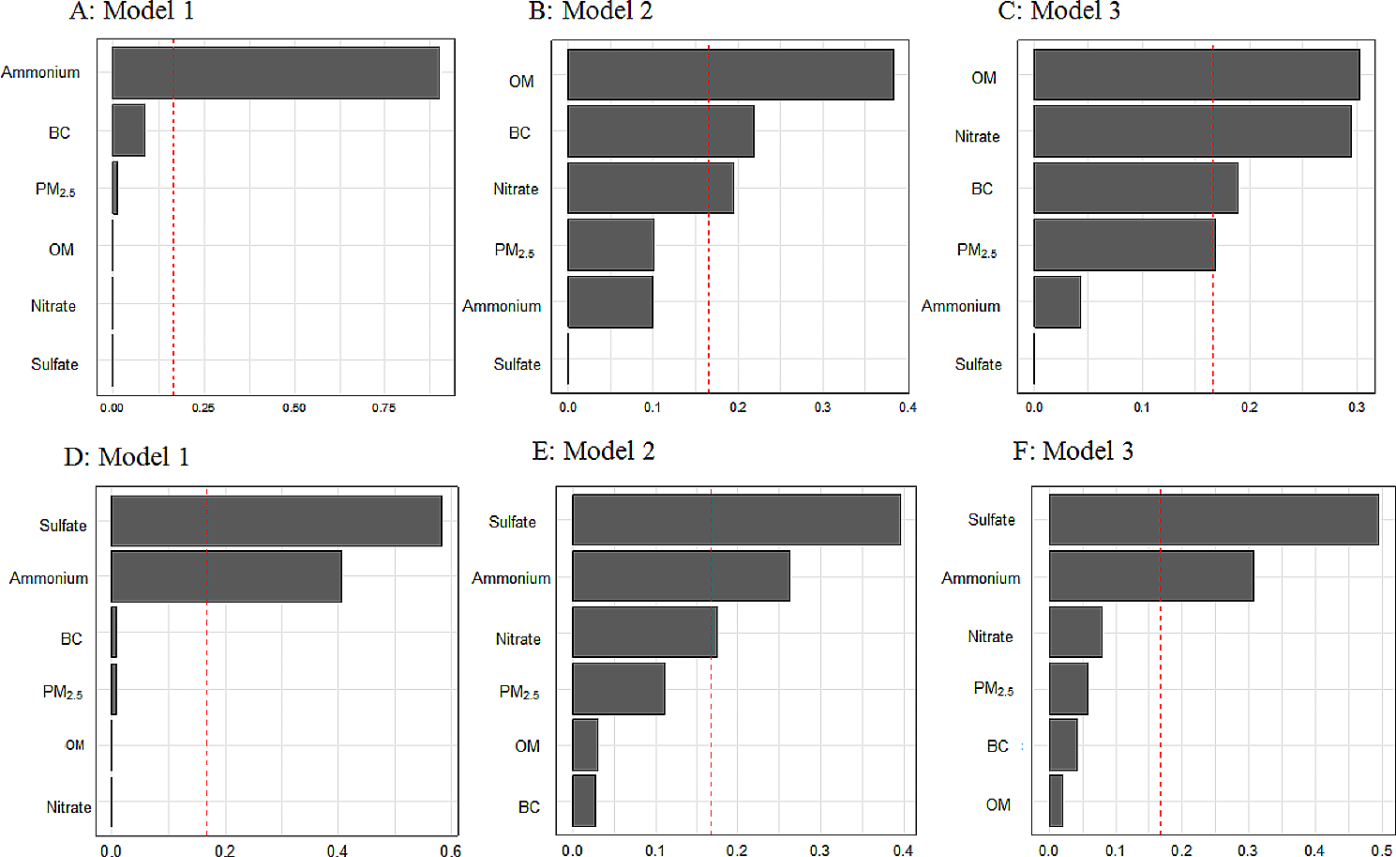
Weight estimations of PM_2.5_ and its components by weighted quantile sum (WQS) method. **A-C**: Associations of multi-pollutant exposure of PM_2.5_ and its components with age at menarche (year). **D-F**: Associations of multi-pollutant exposure of PM_2.5_ and its components with early menarche (< 12 years). Model 1: crude model, adjusted for no covariates. Model 2: adjusted for wave, surveyed district, residency, ethnicity, parental highest education level, physical activity, second-hand smoke. Model 3: adjusted for wave, surveyed district, residency, ethnicity, parental highest education level, physical activity, second-hand smoke, BMI z-score at survey time. PM_2.5_: particulate matter with aerodynamic diameters ≤ 2.5 μm; OM: organic matter; BC: black carbon

Joint exposure to PM_2.5_ and its components was inversely associated with age at menarche (years) in model 1 (β=-0.13, *P* = 0.02), while this association became not significant after further adjustment for potential covariates (Table 4). The estimated weights of PM_2.5_ and its components for age at menarche were shown in Fig. 3A-C. For menarche onset, joint exposure to PM_2.5_ and its components was positively associated with early menarche after adjusting for potential covariates (OR: 1.11, 95% CI: 1.01–1.20, *P* = 0.04 in model 3). Figure 3D-F outlined the estimated weights of the three models. In model 3, the estimated weight of sulfate (0.495) was the largest, followed by ammonium (0.307), nitrate (0.081) and PM_2.5_ (0.057) (Fig. 3F).

**Table 4:** Associations of 1-year multi-pollutant exposure of PM_2.5_ and its components with age at menarche (year) and early menarche (< 12 years) by WQS models

WQS index of the mixture	Age at menarche (year)				Early menarche (< 12 years)	
	β	SE	*P* value		OR (95% CI)	*P* value
Model 1^a^	-0.13	0.05	0.02		1.02 (1.00, 1.06)	0.06
Model 2^b^	-0.19	0.16	0.3		1.11 (1.02, 1.21)	0.03
Model 3^c^	-0.25	0.17	0.1		1.11 (1.01, 1.20)	0.04

WQS: weighted quantile sum; SE: standard error; OR: odds ratio; CI: confidence interval

^a^ Model 1: crude model, adjusted for no covariates

^b^ Model 2: adjusted for wave, surveyed district, residency, ethnicity, parental highest education level, physical activity, second-hand smoke

^c^ Model 3: adjusted for wave, surveyed district, residency, ethnicity, parental highest education level, physical activity, second-hand smoke, BMI z-score at survey time

### Sensitivity analysis

For 2-year exposure before menarche onset: after controlling for potential confounders, higher annual average concentrations of PM_2.5_, sulfate, nitrate and ammonium were consistently linked with decreased age at menarche (all *P <* 0.001, Additional file 1: Table S1) and elevated risk of early menarche (all *P <* 0.05, Additional file 1: Table S2); higher level of joint exposure to PM_2.5_ and its components was associated with higher odds of early menarche (OR: 1.11, 95%CI: 1.01–1.21, Additional file 1: Table S3). For 3-year exposure before menarche onset: in model 3, average exposure to nitrate and ammonium was negatively related to age at menarche (all *P* ≤ 0.001, Additional file 1: Table S1) and positively associated with early menarche (all *P <* 0.05, Additional file 1: Table S2); joint exposure to mixed pollutants was not significantly associated with early menarche (Additional file 1: Table S3). Additionally adjusting for annual average temperature did not change the associations of PM_2.5_ (components) with menarcheal age (Additional file 1: Table S4) and early menarche (Additional file 1: Table S5).

## Discussion

To the best of our knowledge, this is the first population-based epidemiological study investigating the associations of PM_2.5_ components with menarche timing. Using data from a nationally representative cohort, we found that higher pre-menarche exposure to ambient PM_2.5_ and its components (sulfate, nitrate and ammonium) was associated with increased risk of early menarche (< 12 years) among Chinses girls. Furthermore, sulfate might play the most vital role in the relationship between PM_2.5_ and menarche onset.

There is a paucity of published literature examining the relationship between outdoor air pollution and pubertal development. The results from two longitudinal studies were not in line with each other. One study conducted in 437 American girls suggested that higher residential proximity to traffic was associated with earlier pubarche, but not thelarche, as indicated by Tanner stage [29]. While another one revealed contradictory results showing that PM with aerodynamic diameters ≤ 10 μm (PM_10_) exposure in prenatal and infantile periods might delay pubertal onset among girls in Hong Kong, China [16]. Besides, three cross-sectional studies were conducted as well. Jung et al. demonstrated that higher exposure to PM_10_ might decrease the age at menarche, resulting in advanced pubertal development among 639 girls aged 13–17 years from South Korea [12]. Similarly, a research carried out among 1257 women aged 19–25 years living in Poland found that higher level of PM_10_, PM_2.5_ and nitric oxide (NO) was related to younger age at menarche [17]. Nonetheless, results from two German birth cohorts indicated no significant associations between ambient air pollutants (PM_2.5_, PM_10_, NO_2_ and O_3_) and pubertal development determined by estradiol and testosterone levels among children aged 10 years [19]. The inconsistent results of these studies might be due to divergences in study design, population characteristics, assessment of pubertal development, varieties of air pollutants and potential confounders, such as socioeconomic status and exposure metrics.

All of the above studies focused on outdoor air pollutant and puberty-related outcomes. Regarding the exposure to PM_2.5_ on puberty, Wronka [17] and Zhao [19] concluded inconsistent results. In this study, we identified PM_2.5_ as the underlying risk factors for early menarche, which was broadly congruent with Wronka’s study. Notably, the association between PM_2.5_ and puberty onset might vary from exposure level. The average concentration of PM_2.5_ was 53.08 µg/m^3^ in our study, much higher than that in Zhao’s study (14.76 µg/m^3^), which showed no significant association [19]. Furthermore, time windows are relevant to health effects as well [30]. We concentrated on the long-term effect of PM_2.5_ exposure (1 year to several years of exposure), while some studies observed a short-term or medium term of exposure (range from a few days to several months) [18]. In this regard, female adolescents should be aware of the air quality in their living districts and strengthen personal protection to mitigate the detrimental exposure.

While no literature has investigated long-term exposure to ambient PM_2.5_ components on menarche timing, a handful of studies have investigated the effects of their precursors, including nitrogen oxide (NO_x_) and sulfur oxide, albeit with discrepant results [16, 17, 19]. Our study revealed that higher exposure to sulfate, nitrate, and ammonium was associated with early menarche timing in single-pollutant analysis, which was also suggested to make larger contribution in the joint exposure analysis by WQS regression models. Remarkably, sulfate was the most important contributors in the association between PM_2.5_ and early menarche, however, it was not significantly associated with age at menarche (years) in the generalized linear model. The possible explanation might be that the association between sulfate and age at menarche is not strictly linear and this association was much more obvious when the outcome variable was dichotomous.

The biological mechanisms linking ambient air pollution with pubertal development are still unclear. Previous studies have implied that PM may induce oxidative stress, leading to inflammations in respiratory and cardiovascular system [31]. Moreover, they might contain some EDCs that could interact with estrogen receptors and initiate the secretion of gonadotropin releasing hormone, thereby accelerating maturation of hypothalamus and causing earlier puberty [32–34]. It was also shown that PM could play a vital role in hormonal function of the female reproductive system by mimicking normal hormones [35, 36].

However, the relative studies concerning mechanisms of specific PM_2.5_ components on pubertal development are scarce. Majority of sulfate and nitrate in PM_2.5_ originate from the atmospheric oxidation of SO_2_ and NO_x_ emissions, while ammonium is formed by sulfuric or nitric acid reacting with ammonia in the atmosphere [37]. Previous researches inferred that SO_2_ could have suppressive effects on androgens, and it might play crucial roles in inducing systematic inflammation and coagulation [16, 38]. Nitrate in PM_2.5_ might cause oxidative damage and reduce gonadal steroidogenesis, which could affect secretion of sex hormones in turn [39, 40]. Studies on health effects of ammonium indicated that short-term exposure to ammonium was positively associated with inflammatory biomarkers [38]. Nevertheless, the potential mechanisms of its relationship with puberty are not fully understood. Further animal experiments and epidemiological studies are imperative to ascertain the exact mechanism.

In further analysis, we found that both single and joint exposure to ambient PM_2.5_ and its components (1-year and 2-year before menarche onset) increased the risk of early menarche, however, 3-year annual average joint exposure imposed no significant impact on early menarche. This was aligned with study conducted by Jung [12] elucidating that the magnitude of association between PM_10_ and menarche timing was weaker when the exposure period prolonged to 3 years. The potential reason might be that the internal and external factors were constantly changing in a long period, and it was difficult to control the relevant covariates 3 years before menarche onset, leading to inconsistent results. The results of 1-year and 2-year exposure might be worth more concentration in this study.

Several limitations for the current study should be acknowledged. Firstly, the cross-sectional design limited the ability to establish casual inferences, and prospective cohort studies are required for validation. However, we selected 1-year average concentrations of pollutants before menarche onset year as exposure, which roughly ensured the sequence of exposure and outcome. Secondly, the precise timing of exposure could not be verified because the exact date of menarche was not obtained in data from CHNS. And the self-reported age at menarche might cause recall bias. Notwithstanding, the participants were restricted to girls aged 10–17 years with relatively shorter time interval between menarche onset and survey time (mean ± SD: 1.58 ± 1.26 years, 54.3% of participants were within 1 year), and 2-year/3-year average exposure before menarche onset was also considered in the sensitivity analysis. Furthermore, as mother’s menarche timing was not obtained from CHNS dataset, parental genetic factors were not adjusted in the statistic model. We will consider more comprehensive confounders in future study. Finally, measures of air pollutant exposure were calculated according to the residential district without considering the individual exposure patterns (e.g., outdoor activity time, range of motion and usage of protective mask). Further information is necessary to explore these associations at individual level.

Despite the limitations, our study has several strengths. We utilized a nationwide sample from CHNS, the diversity of which in geography, economics and infrastructures could generalize our results to all the female adolescents around China. As individuals are exposed to both PM_2.5_ and its components, we conducted WQS method to examine the association of multi-pollutant exposure with menarche timing as well, making the results more comprehensive. Additionally, many relevant confounders, including physical activity, second-hand smoke exposure at home and BMI z-score, were considered for adjustment simultaneously. Overall, on the basis of robust models we constructed, this study might pave the way for future researches into the associations between PM_2.5_ components and menarche onset among girls.

## Conclusion

The current study suggested that long-term pre-menarche exposure to ambient PM_2.5_ and its components was associated with younger age at menarche and increased risk of early menarche (< 12 years) among Chinese girls. Furthermore, sulfate and ammonium imposed much stronger impacts on menarche onset in the multi-pollutant exposure. As early puberty onset is a crucial risk factor for large quantities of adverse health outcomes in adulthood, this study gained new sights into the promotion of adolescent health from an environmental standpoint. It is of vital importance to control the source of sulfate and ammonium to curb the burden of PM_2.5_-related puberty abnormalities. Longitudinal prospective study with a large sample size and mechanism researches are warranted in the future.

### Electronic supplementary material

Below is the link to the electronic supplementary material.

Supplementary Material 1

## Data Availability

No datasets were generated or analysed during the current study.

## Abbreviations

BC: black carbon
BMI: body mass index
CHNS: China Health and Nutrition Survey
EDCs: endocrine-disrupting chemicals
GLM: generalized linear model
NO: nitric oxide
NO_2_: nitrogen dioxide
NO_x_: nitrogen oxide
O_3_: ozone
OM: organic matter
PM: particulate matter
PM_2.5_: particulate matter with aerodynamic diameters ≤ 2.5 μm
PM_10_: particulate matter with aerodynamic diameters ≤ 10 μm
SO_2_: sulfur dioxide
TAP: Tracking Air Pollution in China
WQS: weighted quantile sum
