# Supplementary material for: Long-term exposure to ambient PM2.5 and its components on menarche timing among Chinese adolescents: evidence from a representative nationwide cohort
Source: BMC Public Health. 2024 Mar 5;24:707. doi: 10.1186/s12889-024-18209-2 (PMC10916212; doi:10.1186/s12889-024-18209-2)
Supplement: Supplementary file 1 — Supplementary Material 1 [file 12889_2024_18209_MOESM1_ESM.docx]

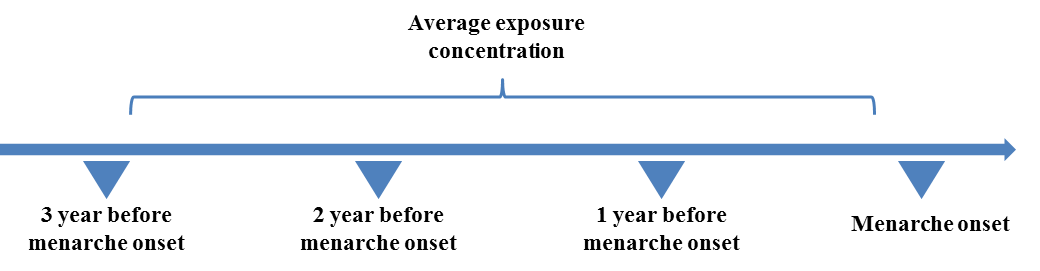


**Figure S1** A schematic representation of exposure periods

**Table S1** Associations of 2-year and 3-year average exposure to PM_2.5_ and its components (per 1 μg/m^3^ increase) with age at menarche (year) by generalized linear regression models ^a^

| Pollutant | Model 1^b^ | | |  | Model 2^c^ | | |  | Model 3^d^ | | |
| --- | --- | --- | --- | --- | --- | --- | --- | --- | --- | --- | --- |
|  | β | SE | *P* value |  | β | SE | *P* value |  | β | SE | *P* value |
| 2-year average (n=807) |  |  |  |  |  |  |  |  |  |  |  |
| PM_2.5_ | -0.003 | 0.003 | 0.3 |  | -0.026 | 0.010 | 0.007 |  | -0.023 | 0.01 | 0.02 |
| Sulfate | -0.023 | 0.014 | 0.1 |  | -0.131 | 0.049 | 0.008 |  | -0.116 | 0.05 | 0.02 |
| Nitrate | -0.016 | 0.012 | 0.2 |  | -0.162 | 0.036 | <0.001 |  | -0.145 | 0.04 | <0.001 |
| Ammonium | -0.036 | 0.018 | 0.046 |  | -0.218 | 0.053 | <0.001 |  | -0.194 | 0.05 | <0.001 |
| BC | -0.046 | 0.058 | 0.4 |  | 0.022 | 0.171 | 0.9 |  | 0.005 | 0.17 | 0.9 |
| OM | -0.014 | 0.012 | 0.2 |  | -0.038 | 0.041 | 0.3 |  | -0.035 | 0.04 | 0.4 |
| 3-year average (n=726) |  |  |  |  |  |  |  |  |  |  |  |
| PM_2.5_ | -0.002 | 0.003 | 0.5 |  | -0.026 | 0.011 | 0.02 |  | -0.020 | 0.011 | 0.07 |
| Sulfate | -0.019 | 0.015 | 0.2 |  | -0.124 | 0.058 | 0.03 |  | -0.095 | 0.057 | 0.09 |
| Nitrate | -0.015 | 0.012 | 0.2 |  | -0.172 | 0.040 | <0.001 |  | -0.148 | 0.040 | <0.001 |
| Ammonium | -0.033 | 0.019 | 0.08 |  | -0.226 | 0.060 | <0.001 |  | -0.190 | 0.059 | 0.001 |
| BC | -0.035 | 0.060 | 0.6 |  | 0.135 | 0.197 | 0.5 |  | 0.148 | 0.193 | 0.4 |
| OM | -0.011 | 0.013 | 0.4 |  | -0.017 | 0.047 | 0.7 |  | -0.007 | 0.047 | 0.9 |

PM: particulate matter; BC: black carbon; OM: organic matter

^a^ Single exposure analysis.

^b^ Model 1: crude model, adjusted for no covariates.

^c^ Model 2: adjusted for wave, surveyed district, residency, ethnicity, parental highest education level, physical activity, second-hand smoke.

^d^ Model 3: adjusted for wave, surveyed district, residency, ethnicity, parental highest education level, physical activity, second-hand smoke, BMI z-score at survey time.

**Table S2** Associations of 2-year and 3-year average exposure to PM2.5 and its components (per 1 μg/m^3^ increase) with early menarche

(< 12 years) by logistic regression models ^a^

| Pollutant | Model 1^b^ | |  | Model 2^c^ | |  | Model 3^d^ | |
| --- | --- | --- | --- | --- | --- | --- | --- | --- |
|  | OR (95% CI) | *P* value |  | OR (95% CI) | *P* value |  | OR (95% CI) | *P* value |
| 2-year average (n=807) |  |  |  |  |  |  |  |  |
| PM_2.5_ | 1.01 (1.00, 1.02) | 0.04 |  | 1.07 (1.02, 1.12) | 0.004 |  | 1.06 (1.01, 1.11) | 0.01 |
| Sulfate | 1.06 (1.00, 1.12) | 0.04 |  | 1.39 (1.11, 1.75) | 0.005 |  | 1.33 (1.06, 1.67) | 0.02 |
| Nitrate | 1.06 (1.01, 1.10) | 0.02 |  | 1.38 (1.17, 1.65) | <0.001 |  | 1.32 (1.11, 1.58) | 0.002 |
| Ammonium | 1.09 (1.02, 1.17) | 0.01 |  | 1.57 (1.22, 2.04) | <0.001 |  | 1.47 (1.14, 1.92) | 0.004 |
| BC | 1.08 (0.86, 1.37) | 0.5 |  | 1.43 (0.69, 2.96) | 0.3 |  | 1.46 (0.70, 3.06) | 0.3 |
| OM | 1.05 (0.99, 1.10) | 0.06 |  | 1.18 (0.99, 1.41) | 0.06 |  | 1.18 (0.98, 1.40) | 0.09 |
| 3-year average (n=726) |  |  |  |  |  |  |  |  |
| PM_2.5_ | 1.01 (0.99, 1.02) | 0.1 |  | 1.06 (1.01, 1.12) | 0.02 |  | 1.05 (0.99, 1.10) | 0.07 |
| Sulfate | 1.05 (0.99, 1.11) | 0.1 |  | 1.33 (1.04, 1.72) | 0.03 |  | 1.24 (0.96, 1.61) | 0.1 |
| Nitrate | 1.05 (1.00, 1.10) | 0.04 |  | 1.35 (1.13, 1.64) | 0.001 |  | 1.28 (1.06, 1.55) | 0.01 |
| Ammonium | 1.08 (1.01, 1.16) | 0.04 |  | 1.50 (1.14, 1.99) | 0.005 |  | 1.37 (1.04, 1.84) | 0.03 |
| BC | 1.06 (0.84, 1.34) | 0.7 |  | 1.27 (0.57, 2.88) | 0.6 |  | 1.22 (0.54, 2.81) | 0.6 |
| OM | 1.04 (0.99, 1.09) | 0.1 |  | 1.16 (0.95, 1.42) | 0.2 |  | 1.13 (0.92, 1.38) | 0.2 |

PM: particulate matter; BC: black carbon; OM: organic matter

^a^ Single exposure analysis.

^b^ Model 1: crude model, adjusted for no covariates.

^c^ Model 2: adjusted for wave, surveyed district, residency, ethnicity, parental highest education level, physical activity, second-hand smoke.

^d^ Model 3: adjusted for wave, surveyed district, residency, ethnicity, parental highest education level, physical activity, second-hand smoke, BMI z-score at survey time.

**Table S3** Associations of 2-year and 3-year multi-pollutant exposure of PM_2.5_ and its components with early menarche (<12 years) by WQS models

|  | 2-year average exposure | |  | 3-year average exposure | |
| --- | --- | --- | --- | --- | --- |
|  | OR (95% CI) | *P* value |  | OR (95% CI) | *P* value |
| Model 1^a^ | 1.03 (0.99, 1.06) | 0.09 |  | 1.03 (1.00, 1.07) | 0.049 |
| Model 2^b^ | 1.12 (1.02, 1.22) | 0.02 |  | 1.09 (0.97, 1.21) | 0.1 |
| Model 3^c^ | 1.11 (1.01, 1.21) | 0.04 |  | 1.06 (0.94, 1.72) | 0.3 |

^a^ Model 1: crude model, adjusted for no covariates.

^b^ Model 2: adjusted for wave, surveyed district, residency, ethnicity, parental highest education level, physical activity, second-hand smoke.

^c^ Model 3: adjusted for wave, surveyed district, residency, ethnicity, parental highest education level, physical activity, second-hand smoke, BMI z-score at survey time.

**Table S4** Associations of 1-year average exposure to PM_2.5_ and its components (per 1 μg/m^3^ increase) with age at menarche (year) by GLM ^a^

| Pollutant |  | Model 1^b^ | | |  | Model 2^c^ | | |
| --- | --- | --- | --- | --- | --- | --- | --- | --- |
|  |  | β | SE | *P* value |  | β | SE | *P* value |
| PM_2.5_ |  | -0.014 | 0.009 | 0.1 |  | -0.013 | 0.009 | 0.1 |
| Sulfate |  | -0.063 | 0.044 | 0.2 |  | -0.061 | 0.043 | 0.2 |
| Nitrate |  | -0.11 | 0.033 | 0.0008 |  | -0.101 | 0.033 | 0.002 |
| Ammonium |  | -0.14 | 0.048 | 0.004 |  | -0.133 | 0.048 | 0.006 |
| BC |  | 0.14 | 0.148 | 0.4 |  | 0.062 | 0.147 | 0.7 |
| OM |  | -0.004 | 0.036 | 0.9 |  | -0.012 | 0.036 | 0.7 |

GLM: generalized linear model; SE: standard error; PM_2.5_: particulate matter with aerodynamic diameters ≤ 2.5μm; BC: black carbon; OM: organic matter

^a^ Single exposure analysis.

^b^ Model 1: adjusted for wave, surveyed district, residency, ethnicity, parental highest education level, physical activity, second-hand smoke, annual average temperature.

^c^ Model 2: adjusted for wave, surveyed district, residency, ethnicity, parental highest education level, physical activity, second-hand smoke, annual average temperature, BMI z-score at survey time.

**Table S5** Associations of 1-year average exposure to PM2.5 and its components (per 1 μg/m^3^ increase) with early menarche (< 12 years) by logistic regression models ^a^

| Pollutant | Model 1^b^ | |  | Model 2^c^ | |
| --- | --- | --- | --- | --- | --- |
|  | OR (95%CI) | *P* value |  | OR (95%CI) | *P* value |
| PM2.5 | 1.05 (1.01, 1.09) | 0.02 |  | 1.05 (1.11, 1.09) | 0.03 |
| Sulfate | 1.25 (1.03, 1.54) | 0.03 |  | 1.24 (1.02, 1.53) | 0.04 |
| Nitrate | 1.27 (1.10, 1.49) | 0.002 |  | 1.24 (1.06, 1.45) | 0.007 |
| Ammonium | 1.38 (1.10, 1.75) | 0.006 |  | 1.34 (1.07, 1.70) | 0.01 |
| BC | 1.15 (0.61, 2.21) | 0.7 |  | 1.29 (0.67, 2.52) | 0.5 |
| OM | 1.11 (0.95, 1.31) | 0.2 |  | 1.12 (0.96, 1.32) | 0.2 |

OR: odds ratio; CI: confidence interval; PM_2.5_: particulate matter with aerodynamic diameters ≤ 2.5μm; BC: black carbon; OM: organic matter

^a^ Single exposure analysis.

^b^ Model 1: adjusted for wave, surveyed district, residency, ethnicity, parental highest education level, physical activity, second-hand smoke, annual average temperature.

^c^ Model 2: adjusted for wave, surveyed district, residency, ethnicity, parental highest education level, physical activity, second-hand smoke, annual average temperature, BMI z-score at survey time.
